# Updates on the Morphometric Characterization of Indian Pangolin (*Manis crassicaudata*) in Sri Lanka

**DOI:** 10.3390/ani11010025

**Published:** 2020-12-25

**Authors:** Hirusha Randimal Algewatta, Priyan Perera, Hasitha Karawita, Nihal Dayawansa, Dinushika Manawadu, Malith Liyanage

**Affiliations:** 1 Department of Forestry and Environmental Science, University of Sri Jayewardenepura, Nugegoda 10250, Sri Lanka; hirusharandimal.tmp@sjp.ac.lk (H.R.A.); hasithakarawita@sjp.ac.lk (H.K.); 2 IUCN SSC Pangolin Specialist Group, C/o Zoological Society of London, Regent’s Park, London NW1 4RY, UK; 3 College of Science, Health, Engineering, and Education, Environmental and Conservation Sciences, Murdoch University, South Street, Perth, WA 6150, Australia; 4 Department of Zoology and Environment Sciences, University of Colombo, Colombo 0070, Sri Lanka; nihal.dayawansa@sci.cmb.ac.lk; 5 Department of National Zoological Gardens, Dehiwala 10350, Sri Lanka; dinushikam145@yahoo.com (D.M.); malithliyanageliyanage@gmail.com (M.L.)

**Keywords:** morphometry, body weight, scales, sexual dimorphism, body length predictor

## Abstract

**Simple Summary:**

The Indian pangolin (*Manis crassicaudata*) is one of the eight extant pangolin species in the world, listed as “endangered” by the International Union for Conservation of Nature (IUCN). The species is native to the Indian subcontinent. The Indian pangolin population in Sri Lanka is geographically isolated from the mainland population and therefore, could possess morphological adaptations to the unique environmental conditions in the island. However, an accurate description of the morphological features has not been attempted for Indian pangolins occurring on the island. This study described the morphological features of Indian pangolins based on observations made on 27 specimens from Sri Lanka. The adult male Indian pangolin measures between 137 and 177 cm from the snout to the tip of the tail and weighs between 20.4 and 48.8 kg. The largest Indian pangolin recorded so far from any of its range counties, weighing 48.76 kg and measuring 176.8 cm in total body length was recorded in this study. Indian pangolins recorded from Sri Lanka on average bear 511 ± 21 scales. Three major scale morph/types are further described.

**Abstract:**

An accurate morphological description and analysis based on reliable data are unavailable for the geographically isolated population of *M. crassicaudata* in Sri Lanka. This study provides the most updated morphological description of *M. crassicaudata* with special reference to body measurements directly obtained from 27 specimens collected island-wide. Morphological parameters were recorded under three age classes that were defined based on their body weight (BW) and total body length (TBL); juvenile (BW: <4.3 kg TBL: <56.0 cm), subadult (BW: 4.3–7.3 kg TBL: 56–101 cm), and adult (BW: >7.3 kg TBL: >101 cm) and gender to reveal sexual dimorphism based on morphometric parameters. The TBL of adult males ranged between 137 and 177 cm while body weight ranged between 20.4 and 48.8 kg. The average count of body scales was 511 ± 21. The body scales were found arranged in 13 longitudinal rows with the highest number of scales observed on the vertebral scale row (16 ± 1). Three major scale morphs were identified; broad rhombic scales, elongated kite-shaped scales, and folded shaped scales. Broad rhombic shaped scales was the dominant scale type (80.49%) on the body (405 ± 7). The tail-length to body-length ratio of an Indian pangolin was 0.87. The tail length of an Indian pangolin is a reliable predictor of the TBL and has potential implications in quick field data gathering.

## 1. Introduction

The Indian pangolin (*Manis crassicaudata*, Geoffroy 1803), is a solitary, elusive, and predominantly nocturnal mammal native to South Asia [1]. It shows a wide distribution across South Asia, including eastern Pakistan, Sri Lanka, Nepal, and India [2,3]. The Indian pangolin is the only pangolin species occurring in Sri Lanka, and the species is distributed from coastal habitats up to 1850 m above sea level, covering most parts of the country [1,4]. With its behavioral plasticity, the Indian pangolin can inhabit an array of natural and human-modified habitats such as tropical lowland rain forests, dry-mixed evergreen forests, submontane to montane forests, scrublands, croplands such as rubber, oil palm, and tea, as well as rural home gardens [5,6]. Hunting, poaching, and trafficking, primarily driven by the demand for its keratinous scales and meat, have made the Indian pangolin threatened across its range [2]. Despite being identified as “endangered” globally and nationally, limited studies have been conducted on Indian pangolins found in Sri Lanka [5,7,8], and the lack of scientific information on the species is a major impediment in the conservation of Indian pangolins [9].

Geographic isolation is widely accepted as a key contributor to divergent evolution, leading to unique phenotypes of a species [10]. Different phenotypes evolve as adaptations to local environmental differences [11]. Following the separation of Sri Lanka from Gondwana in the late Miocene, the new environmental conditions in Sri Lanka provided a diverse topographic, climatic, and biotic stage for the mammals mostly of Indian-Indochinese origin to develop unique adaptations [12]. For instance, a significant genetic differentiation has been observed in the Asian elephant (Elephas maximus) populations between the mainland and Sri Lanka, as well as among northern, mid-latitude, and southern regions of Sri Lanka leading to subspecies level divergence of *Elephas maximus maximus* [13]. The literature reports evidence for intraspecific variations in morphometrics of the Indian pangolin across its range [14,15,16]. Although the Indian pangolin occurs in most parts of South Asia, the Indian pangolin population in Sri Lanka may be of special interest owing to its geographical isolation from the mainland population [5,9]. However, morphometric variations of the Indian pangolin in Sri Lanka remain poorly understood.

General morphological and anatomical features of the Indian pangolin have been described in the literature [9,14,17,18,19,20,21,22,23]. However, detailed descriptions of the morphology and anatomy of wild populations of Indian pangolin are not well documented across its range [14]. The Indian pangolin is described as a medium-sized mammal with an elongated body and tail covered with keratinous scales [19]. An adult Indian pangolin may typically weigh between 8 and 16 kg and can measure up to 148 cm in length from snout to the tip of the tail [2]. Known also as the “Thick-tailed Pangolin”, *M. crassicaudata* has a dorsally flat, thick, and muscular prehensile tail, which is almost wide as the body’s posterior at the proximal end, and gradually tapering towards the distal end [19,22]. The tail can represent 39–54% of the total body length [2].

The Indian pangolin is sexually dimorphic, and a fully grown adult male is much larger and heavier than a female of the same age [22]. However, the published morphoanatomical descriptions on the sexual dimorphism of the species may lack accuracy and comprehensiveness as they are based on observations made on a limited number of specimens from restricted geographies. On the other hand, one of the major obstacles to the study of Indian pangolins in the wild is locating them, especially owing to their nocturnal behavior [9]. As such, indirect field evidence and camera trapping are commonly employed [24]. However, camera trapping allows fewer opportunities to identify individuals and often fails to generate necessary information such as the individual’s gender and growth stage. Therefore, a detailed description of visible morphological features concerning sexual dimorphism of Indian pangolin can be helpful in such field studies.

Knowledge of the age of individual animals is central to understanding the structures of populations and demographics, and for the successful implementation of conservation strategies for the mammals [24,25,26]. In literature, the age class/growth stage of pangolins has been either roughly estimated or crudely determined based on morphometric measurements [3,27]. For instance, Irshad et al. [14] categorized Indian pangolins into three age classes: juveniles (≤2.5 kg, 40–65 cm), subadults (2.51–8 kg, 66–120 cm) and adults (≥8 kg, ≥120 cm) based on the positive correlation observed between the body weight and total body length of Indian pangolins. Describing the correlations among different morphometric measurements can also provide useful insights into the age of an individual animal [26,28,29]. Furthermore, morphological features such as claw length and shape can further vary among different species of pangolins depending on the use [30]. For instance, the claw length and shape of arboreal pangolin species may differ from ground-dwelling pangolin species. Similarly, intraspecific variations in claw characteristics are also possible as adaptations to the unique environmental conditions the pangolins occupy.

Only a few studies thus far have specifically examined the morphoanatomical characteristics of the Indian pangolin [14,19,22,31,32]. An accurate morphological description and analysis based on reliable data are unavailable in the literature for Indian pangolins occurring in Sri Lanka. An accurate morphoanatomical description of the geographically isolated population of *M. crassicaudata* in Sri Lanka would provide the foundation for studies examining the possible genetic differentiation from the mainland population. Furthermore, improved knowledge of morphoanatomical characteristics of the Indian pangolin will have essential implications in distinguishing the species, growth stage, gender and tracing the origin of the specimens in illegal international trade. Hence, this study described the morphological features of *M. crassicaudata* with special reference to the shape, frequency, and orientation of body scales and morphology of claws based on observations made on 27 specimens, and further explored the relationship between different morphometric measurements.

## 2. Materials and Methods

### 2.1. Specimen Selection for the Study

**Figure 1:**
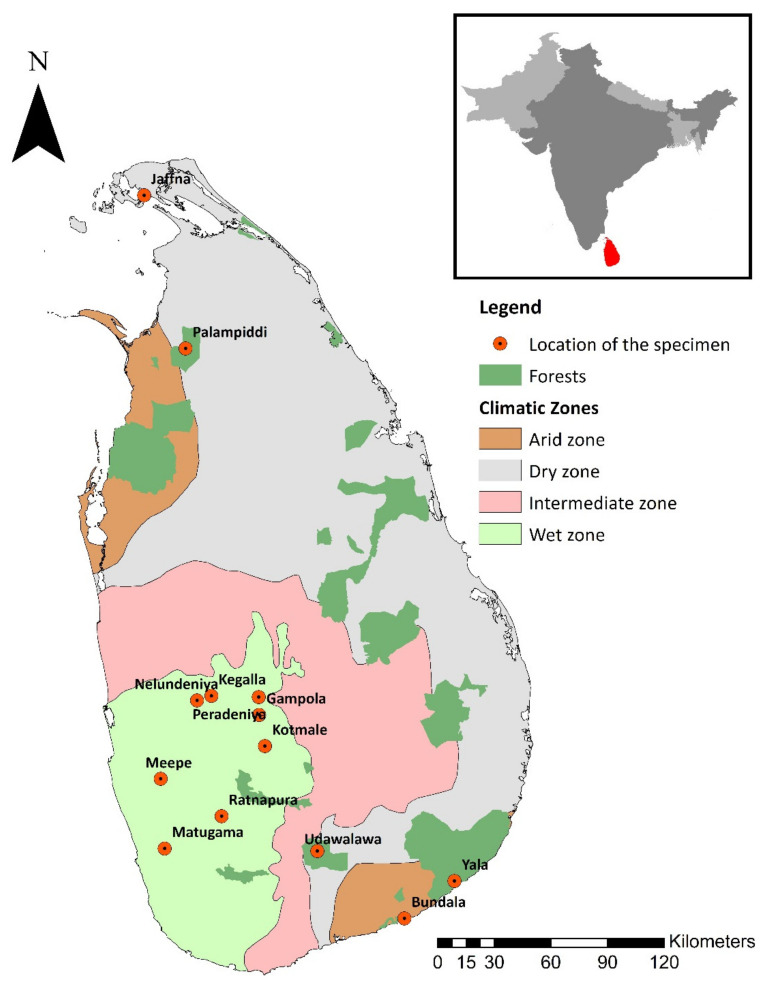
The origin/locations of pangolin specimens observed.

In this study, data on the selected morphometric measurements were gathered from live specimens, fresh carcasses, and museum specimens of *M. crassicaudata,* which is a common sampling approach adopted in similar studies [16,32,33]. Museum specimens were obtained from the National Museum Colombo, Wildlife Training Center Giritale, and Interpretation centers of the Yala National Park and Galway’s Land National Park in Sri Lanka. Dead/preserved specimens were obtained from the Department of Basic Veterinary Science of the Faculty of Veterinary Science of the University of Peradeniya, the National Zoological Gardens Pinnawala, the Postgraduate Institute of Archeology of the University of Kelaniya (PGIAR) and the Wildlife Rehabilitation Center, Kilinochchi in Sri Lanka (Table 1). Morphometric data of living specimens were collected from both field observations and captive individuals at the National Zoological Gardens Pinnawala. A total of 27 specimens were investigated, and their details are summarized in Table 1. The origins of the specimens examined in this study were from dry and wet climatic zones of the country (Figure 1).

In the context of this study, gathering morphometric measurements from museum specimens and live pangolins did not require ethical clearance from an ethical review committee in Sri Lanka as nonintrusive sampling methods were used. The necessary approval to gather morphometric data from live pangolins using the established protocols [34] was obtained from the National Research Committee of the Department of Wildlife Conservation, Sri Lanka (Approval Reference: WL/3/2/41/19). The National Research Committee of the Department of Wildlife Conservation, Sri Lanka has waived the need for ethical clearance for this study. Furthermore, the funding institution for this study (University of Sri Jayewardenepura, Sri Lanka) has a policy of funding and approving only the research projects that satisfactorily meet ethical considerations. Based on the nature of the information sought and methods used [34], the Research Council of the University of Sri Jayewardenepura, Sri Lanka has determined that this research does not require ethical clearance and thus, the study was approved (Research grant/Registration No: ASP/01/RE/SCI/2017/14).

### 2.2. Recording of Body Measurements

**Figure 2:**
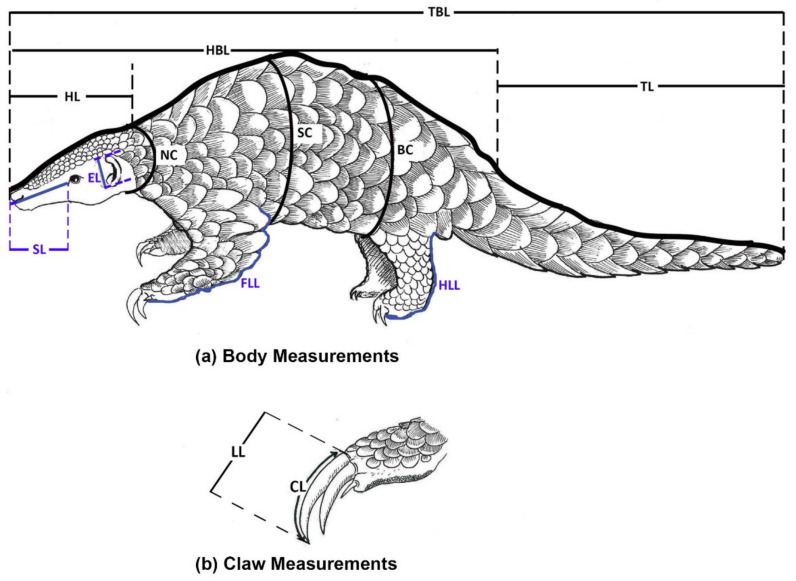
Morphometric parameters measured on *M. crassicaudata:* (**a**) body measurements and (**b**) claw measurements (see Perera et al. [34] for a detailed description of morphometric measurements).

Out of the 27 specimens, only 22 specimens (7 live specimens, 4 fresh carcasses, and 11 complete museum specimens) were used to gather morphometric measurements. The rest of the five museum specimens were used only to measure the morphological characteristics of scales since the specimens were too old and incomplete. Following the protocols described in Perera et al. [34], thirteen different morphometric parameters were recorded from specimens (Table 2, Figure 2a). The body weights of live and fresh carcasses were recorded using a portable electric balance (WeiHeng WH-A08). The dead specimens were stored in deep freezers within less than 18 h of death, hence remained in more or less original condition. Weight measurements were not obtained from museum specimens. The external measurements of the body were taken using a flexible measuring tape. The ear length was measured using a digital Vernier caliper (Mitutoyo 500-196-30).

In this study, nonintrusive methods were used to gather morphometric measurements, and standard anesthetizing procedures were not used. Handling of live pangolins was done following the protocols described in Perera et al. [34]. Measurements were taken while the animal was held up or after placing the animal on a flat surface (animal placed in a physiological recumbent position) where possible.

### 2.3. Measurements of Claws

The numbering of claws and recording of claw measurements followed the protocols described in Perera et al. [34]. Only the claw measurements of forelimbs were recorded. The linear length (LL) and curvature length (CL) [40] of claws were measured using a digital Vernier caliper and a flexible measuring tape respectively (Figure 2b). The straight carapace and curved length of the claw along its external perimeter were used to compute the claw index (CLI) as described in Alarcos et al. [40].

$$\mathrm{Claw}\;\mathrm{Index}\left(\mathrm{CLI}\right)=\left[\frac{\mathrm{CL}-\mathrm{LL}}{\mathrm{CL}+\mathrm{LL}}\right]\times 100$$

$$\mathrm{Linear}\;\mathrm{to}\;\mathrm{curvature}\;\mathrm{length}\;\mathrm{ratio}\;\left(\mathrm{LCR}\right)=\left[\frac{\mathrm{LL}}{\mathrm{CL}}\right]\times 100$$

### 2.4. Scale Counts and Measurements

**Figure 3:**
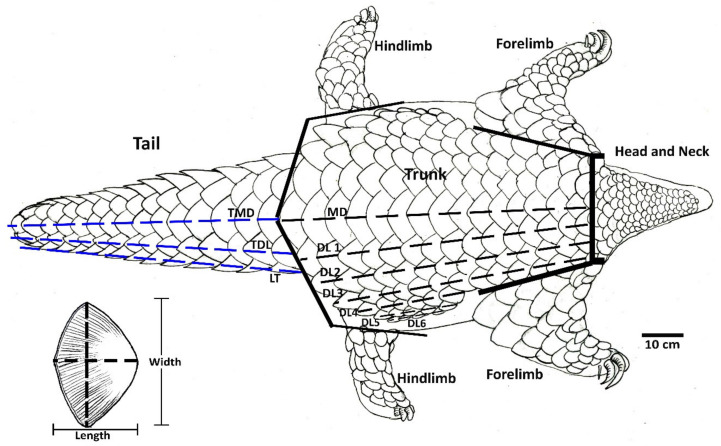
Definition of body regions and scale rows to perform scale counts; MD = mediodorsal row/vertebral row, DL 1-6 = trunk dorsolateral rows, TMD = tail mediodorsal row, TDL= tail dorsolateral row, LT = lateral row (adapted from Perera et al. [34]).

Dorsal, lateral, and ventral surfaces of each specimen were photographed using a Canon EOS 70D Digital Single Lens Reflex (DSLR) camera to ensure that all scales were captured in photographs for counting. Comprehensive scale counts were performed manually, as well as using photographs visualized in Adobe Photoshop and ImageJ software. Scale counts were performed separately by body region, i.e., head, forelimbs, hind limbs, trunk, and tail (see Perera et al. [34] for detailed scale counting protocols for Indian pangolin). Scale counts on trunk and tail regions were performed along longitudinal rows identified during preliminary observations and defined as in Figure 3. In cases where a scale was missing, but it could be determined with confidence that a scale was once present, i.e., there was an obvious scale bed, it was included in the scale count [16].

The length and width of scales were measured using a digital vernier caliper (Mitutoyo 500-196-30). The distance between the two widest points of a scale was taken as the scale width and the distance between the base and the tip of the scale was considered as the length of the scale (Figure 3). Measurements of the scales on hind legs and lower parts of the forelegs were not considered in this study. Grooves on the scales were further counted manually and using photographs visualized in ImageJ software. Scales with damaged or worn-out surfaces were excluded from the detailed analysis [41].

### 2.5. Analysis of Data

Both descriptive and inferential statistical methods were used in data analysis as the collected data included qualitative and quantitative information, The K-mean cluster analysis was employed to group the specimens into juvenile, subadult, and adult categories based on total body length and circumference of the shoulder. The total body length was used as it is a distinct parameter positively correlated to age [42,43,44]. The circumference of the shoulder was selected as it is known to have a positive correlation with body weight and age [38,43,45,46,47]. Body weight was not used as a clustering variable because weight measurements were available for only 13 specimens.

Comparisons of selected morphometric measurements among different age groups were done using the Kruskal−Wallis test. Chi-square test was employed to explore the association between gender and the tail-to-body length ratio. An independent sample *t*-test was further used to identify significant differences in the measured claw parameters and scale counts between males and females. The data gathered for all variables were tested for normality using the Shapiro−Wilk test since the test has a higher power, and is often recommended for small sample sizes [48,49]. The Pearson’s Correlation test was used to explore the correlations between the easily measurable morphometric parameters. Relationships between morphometric parameters were further explored using linear regression models. All analyses were performed using IBM SPSS Statistics (18.0) software package.

## 3. Results

### 3.1. Morphometric Descriptions of Specimens

The individual morphometric measurements recorded from 22 specimens are summarized in Table 3. The specimens observed included 15 males and 7 females. The recorded morphometric measurements showed a wide variability among the specimens (Table 3). The total body length of the studied female specimens varied from 46.3 cm to 110.7 cm. The largest female specimen weighed 7.33 kg. The largest male specimen weighed 48.76kg and measured 176.8 cm in total length.

### 3.2. Classification of Specimens into Age Classes

Adopting published ranges of morphometric measurements for different age classes in the literature [14,50,51] could not be justified due to the greater variability of the recorded parameters in the studied specimens, The K-means cluster analysis which used the total body length and shoulder circumference as clustering variables segregated the specimens into three distinct groups. Based on the mean values and ranges of the two clustering variables, the three groups were described as juvenile, subadult, and adult (Table 3). Expert knowledge was further used to verify final cluster memberships. Accordingly, morphometric measurements of specimens belonging to each age class are reported in Table 4. Kruskal−Wallis tests also confirmed significant differences in the measured morphometric parameters among the three age classes (*p* < 0.05, *α* = 0.05). The tail-to-body length ratio based on all observed specimens was 0.87 and did not vary across age categories. The tail-to-body length ratio of male and female specimens were 0.84 and 0.93, respectively. However, the Chi-square test revealed no statistically significant association between gender and the tail-to-body length ratio (*Χ^2^* = 21.0, *p* = 0.397).

### 3.3. Relationships among Selected Morphometric Measurements

The Shapiro−Wilk test revealed that all variables were normally distributed (*p* < 0.01, *α* = 0.05). As the ages of the specimens were unavailable, total length and body weight were used as indicators of the approximate growth stage. According to Pearson Correlation test results, all tested morphometric parameters showed statistically significant correlations with tail length having the highest correlation with total body length (*r* = 0.973) followed by shoulder circumference (*r* = 0.964), body circumference (*r* = 0.961) and body length (*r* = 0.940).

#### 3.3.1. Association between Total Body Length (TBL) and Tail Length (TL)

**Figure 4:**
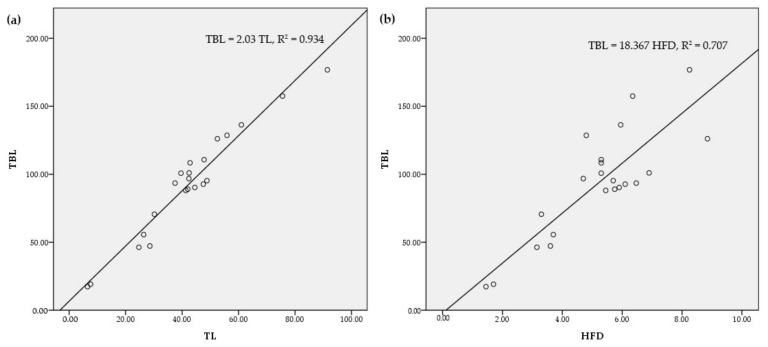
The relationships between (**a**) tail length (TL) and total body length (TBL) (**b**) hindfoot diameter (HFD) and total body length (TBL) for the Indian pangolin.

As all morphometric parameters showed statistically significant correlations with the TBL, linear regression with forward selection was used to explore the relationships. Accordingly, the regression model (1) was derived (*F* = 331.12, *p* = 0.001) as the best and parsimonious model (R^2^ = 0.943, Figure 4a).

(1) TBL=2.03×TL

The derived model was validated with the morphometric dataset reported for Indian pangolin specimens from Pakistan observed by Irshad et al. [14]. The model performed well (R^2^ = 0.774) with residuals evenly distributed. Hence, tail length can be considered a reliable predictor of the total body length of an Indian pangolin.

#### 3.3.2. Association between Total Body Length (TBL) and Hindfoot Diameter (HFD)

It was envisaged that HFD could be used as an indicator of the TBL of a pangolin so that the relationship can be used in field identification purposes. The HFD had a significant positive correlation with TBL (*r* = 0.85, *p* = 0.001). Accordingly, the regression model (2) to predict the TBL using HFD as the predictor yielded a statistically significant model (*F* = 49.95, *p* = 0.001) with R^2^ = 0.707 (Figure 4b).

(2) TBL=1.83×HFD

#### 3.3.3. Association between Body Weight and Other Body Measurements

The Pearson’s Correlation Test results further showed significant positive correlations between body weight and other body measurements with snout length (*r* = 0.970) showing the highest correlation followed by tail length (*r =* 0.919), neck circumference (*r =* 0.918), and head length (*r =* 0.915).

### 3.4. Comparison of Claw Measurements

**Figure 5:**
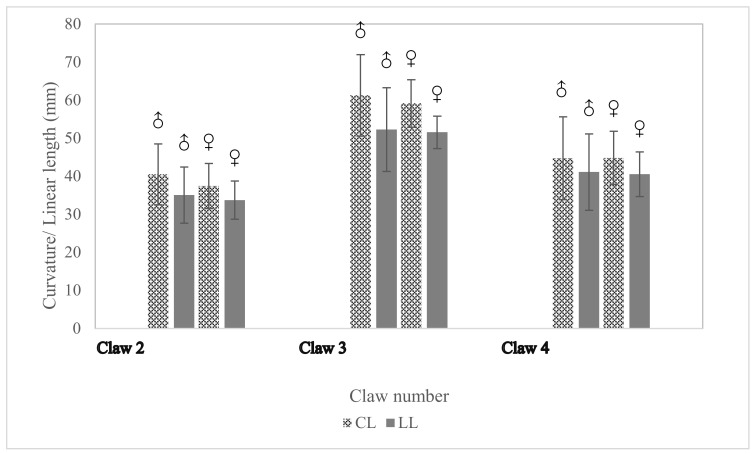
Comparison of the curvature length and linear length claw parameter of males and females belonging to each age class of Indian pangolins (♂: Males, ♀: Females).

The forelimb claw measurements were examined to understand the sexual dimorphism in subadult and adult specimens of Indian pangolins (*n* = 16). For this study, only the three middle claws (2nd, 3rd, and 4th) were measured as the 1st and 5th claw developments were not prominent. Mean values of curvature length (CL) and linear length (LL) of the three middle claws of males and females are shown in Figure 5.

The highest curvature and linear lengths were observed on the 3rd claw. No statistically significant differences in CL, LL, LCI, and LCR were observed between males and females for the 3rd claw (*p* > 0.05).

### 3.5. Scale Morphometry

#### 3.5.1. Scale Frequency

Scales were present on the head, neck (dorsal and lateral sides), trunk (dorsal and lateral), tail (dorsal, ventral, lateral sides), and lateral/outer sides of the hind and forelimbs of the Indian pangolin. Based on the scale counts of 25 specimens, an Indian pangolin on average possesses 511 scales. The average number of scales on different body regions are reported in Table 5. The highest number of scales and the largest scales were observed in the trunk region. Scales present on the head were the smallest. The average number of scales on a juvenile, subadult, and adult specimen was 507 ± 7, 509 ± 20, and 514 ± 7 respectively, which did not change significantly between the different age classes.

#### 3.5.2. Scale Arrangement on Trunk and Tail

It was observed that the body scales of an Indian pangolin were arranged along thirteen longitudinal rows, covering dorsal and ventral sides of the body with a virtual median line of scales oriented along the craniocaudal axis of the body. The vertebral row on average contained 16 ± 1 scales. Dorsolateral rows 1 to 6 (to either side of the vertebral row) contained 15 ± 1, 14 ± 1, 13 ± 1, 7 ± 1, 5, and 4 scales, respectively. The middle row of the tail possessed 15 ± 1 scales, while the dorsolateral and lateral rows had 15 ± 1 scales on each. Scales present on the ventral side of the tail were arranged in 4 rows, but the rows were not distinguishable towards the proximal end of the tail. A terminal scale was present on the ventral surface of the tail. The scale frequencies in each row (body and tail) did not vary among age classes or gender (Table 6). Independent sample *t*-tests further yielded no statistically significant differences in scale frequencies on each row between male and female specimens (*p* > 0.05).

**Figure 6:**
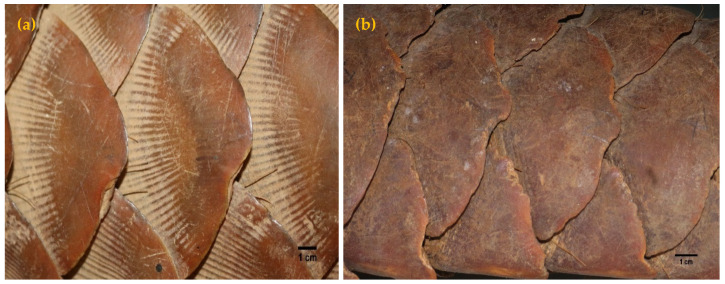
Scale groves of (**a**) adult female and (**b**) adult male Indian pangolins. Groves are largely absent or less visible on the scales of adult males.

Scale orientation on the head, forelimbs, trunk, and tail was parallel to the craniocaudal/longitudinal axis, while the scales on the hind-limbs were oriented vertical to the longitudinal axis. Grooves were present on all scales except those in the head region. Scale grooves were present in specimens of all age groups. However, they were less prominent in adult male specimens (Figure 6).

#### 3.5.3. Scale Types/Morphology

Three main scale morphs [36] were described based on the observations made on Indian pangolin specimens. The three scale morphs are broad rhombic/scapular shaped scales, elongated kite-shaped scales, and folded shaped scales. All scale morphs have distinct features and can be identified visually as well as parametrically. Based on the physical observations and morphometric measurements made on the scales of the Indian pangolin specimens, the three types of scales are described in Table 7.

## 4. Discussion

There is a dearth of accurate scientific information on the morphological features of *M. crassicaudata* in Sri Lanka, except for a few early attempts of morphological descriptions based on limited observations [4,32,52]. This study provided the baseline data on selected body measurements for different sex and age categories of *M. crassicaudata* specimens from Sri Lanka. According to literature, an adult Indian pangolin can weigh between 8 and 16 kg and the total body length could reach about 148 cm [2], however, there are exceptions. For instance, the largest specimen recorded from Pakistan weighed 20 kg and the total body length of the specimen was 147.3 cm [14]. Further, an adult male specimen weighing 32.2 kg and measuring 170 cm in total body length had been recorded from Rajasthan, India [23]. This study recorded possibly the largest male Indian pangolin specimens compared to those documented from India and Pakistan. The two specimens of Indian pangolin recorded from the Mannar District of Sri Lanka weighed 48.76 kg and 34.15 kg while the total body lengths of the specimens were 176.8 cm and 157.5 cm, respectively. The novel observations made in this study on the geographically isolated population of *M. crassicaudata* in Sri Lanka provide new insights into the morphometric variations of the species across its range, and further suggest amendments to the maximum growth of a male Indian pangolin.

Defining the age structure is a fundamental element of population ecology as the study of age-specific survival, mortality, and recruitment rates require the accurate age classification of individual study animals [53]. Three distinct age categories have been often used to compare the morpho-anatomy of mammals; juvenile, subadult, and adult, defined based on body weight and total length [14,27]. Since body weight measurements were available for only live animals and fresh carcasses, total body length and circumference of the shoulder of the animal were used to classify studied specimens into three age categories: juvenile (≤4.3 kg, ≤56.0 cm), subadult (4.3–7.3 kg, 56–101 cm), and adult (≥7.3 kg, ≥101 cm). The body weight and total body length measurement ranges of each age category showed minor deviations from the same measurement ranges reported by Irshad et al. [14] for *M. crassicaudata* specimens from Pakistan.

In this study, the tail-to-body-length ratio observed for the specimens from Sri Lanka was 0.87, which is comparable with the observations of Irshad et al. [14]. Prater [21] and Roberts [22] have reported the same ratio to be 70:30 for *M. crassicaudata,* which is significantly different from the observations in this study. However, these discrepancies may be partially due to the different morphometric measurement protocols used in different studies. The comprehensive and detailed morphometric measurement protocols used in this study are more-or-less comparable with Irshad et al. [14], thus allowing a meaningful comparison.

Although this study attempted to describe the sexual dimorphism of Indian pangolin using the measured morphometric parameters, significant differences between genders in terms of measured parameters were not evident. This may possibly be due to the lack of representation of adult female and male animals in the studied sample. Though the data presented in this study is not representative enough to support the observations in the literature that male Indian pangolins are substantially larger than females of the same age group [3,14,22,52], the research team’s field observations over the past six years buttress this notion. The claw features/parameters were not found to be suitable indicators to distinguish male and female specimens as there were no statistically significant differences observed between genders.

The behavioral plasticity of the Indian pangolin has enabled it to inhabit an array of natural and human-modified landscapes [1]. It has been reported that Indian pangolins exhibit different behavioral adaptations in lowland wet zone habitats and dry zone habitats in Sri Lanka [7]. The specific habitat an individual occupies can further affect the wearing of the claws and scales [7,8,22]. The Indian pangolin specimens observed in this study to record morphometric measurements were from different geographical locations and climatic zones of the country, thus representing different metapopulations. However, the low number of specimens from different geographies did not allow a meaningful comparison of morphological variations among individuals from different geographies.

It has been reported that there are interspecific and intraspecific variations in scale frequency among pangolin species [16]. Hence the accurate description of scale frequency of the Indian pangolin along with knowledge of intraspecific variations in scale frequency and scale morphology will be helpful in species identification in illegal trading, and to estimate the number of individuals of a pangolin species in a given seizure [16,54]. According to Mahmood et al. [3], there are intraspecific variations in total scale numbers in the Indian pangolin, ranging from ~440 to 530. Mohapatra et al. [55] reported the scale frequency of an Indian pangolin to vary from 444 to 519 (474 ± 22, *n* = 8). Ullmann et al. [16] determined that an Indian pangolin on average bears 495 ± 24 (*n* = 9) scales. Based on observations made on 25 specimens, this study reports the average scale frequency of an Indian pangolin to be 511 ± 21. Irshad et al. [14] further reported scale counts by body regions, and accordingly, the tail region of an Indian pangolin contains 82 ± 5 scales. This study reported a much higher scale frequency in the tail region (130 ± 7) while the scale counts on the head, trunk, and limbs did not substantially deviate between the specimens from two geographies. Although different studies in the literature have likely employed different scale counting protocols, the results of the present study are consistent with the literature. Thirteen longitudinal scale rows were identified in all specimens observed in this study, though the literature suggests that the number of longitudinal scale rows can vary from 11 to 13 [4,56]. Nonetheless, the authors believe that the information reported in this study is likely to be the most accurate owing to the larger sample size utilized. Results of this study further support the notion that there is intraspecific variation in total scale frequency in the Indian pangolin.

Establishing relationships between different body measurements and the weight/size of the animal has broad implications in the evaluation of the general health of the population and approximation of the size of individuals through indirect field observations such as footprints and camera trap photographs. Such relationships have been derived for large mammals such as elephants, using heart girth, height at the withers, body length, and foot-pad circumference as vital measurements [57]. In this study, all tested morphometric parameters showed statistically significant correlations with the total body length and weight as in the case of other mammals [26,43,45,46,47,57]. According to regression models developed in this study, the tail length and hindfoot diameter were found to be reliable predictors of total body length. Such relationships may be useful in future studies directed at developing models to determine the body size of pangolins recorded in camera trap photographs. However, it should be mentioned that although there is a significant relationship between hind-foot diameter and total body length for Indian pangolin, the model may require further improvement with a larger sample to be used in field estimations.

## 5. Conclusions

The Indian pangolin shows intraspecific variations in morphometric measurements. Total body length and body weight can be used to classify Indian pangolin into three main age classes: juveniles, subadults, and adults. The new upper limit of body weight and total body length of a male Indian pangolin are 48.76 kg and 176.8 cm, respectively. The largest female specimen weighed 7.33 kg, however, this does not represent the true upper limit of body weight in females as it was observed for a subadult. An Indian pangolin on average has 511 ± 21 scales with broad rhombic scales accounting for 80.49% of the total scales, while elongated kite-shaped and folded-shaped scales accounting for 13.54% and 5.97%, respectively. All measured morphometric parameters showed statistically significant correlations with the total body length with the tail length having the highest correlation with total body length. The tail length of an Indian pangolin is a reliable predictor of the total body length. The total body length can be approximated using hind-foot diameter. The novel baseline data on selected morphometric measurements for different sex and age categories of *M. crassicaudata* specimens from Sri Lanka will be highly useful in future ecological studies on the species where it is necessary to report results based on the species’ sex and age categories. The frequency and orientation of body scales and morphology of claws were not useful in describing the sexual dimorphism of the Indian pangolin.

## Figures and Tables

**Table 1:** Specimens of *M. crassicaudata* investigated in the study.

Specimen Code	Location	Sex	Type	Year of Record
NB1	Pinnawala	Female	Fresh carcass	2018
NB2	Pinnawala	Male	Fresh carcass	2018
PGIAR	Roonakanda	Male	Preserved	2017
KL 1	Palampiddi (Mannar)	Male	Fresh carcass	2019
KL 2	Palampiddi (Mannar)	Male	Fresh carcass	2019
RS	Kegalle	Female	Live	2018
DV	Pinnawala	Female	Live	2019
HY	Yakkalamulla	Male	Live	2019
Zoo RS	Nelundeniya	Female	Live	2019
MP 1	Meepe	Male	Live	2019
MP 2	Meepe	Male	Live	2019
RG	Raigama	Male	Live	2020
NHMC86	Rathnapura	Female	Museum	1953
NHMC86A	Mathugama	Male	Museum	1928
NHMC86C	Rathnapura	Female	Museum	1934
NHMC86E	Jaffna	Female	Museum	1902
NHMC86F	Unknown	Female	Museum	Unknown
GWM-M34	Udawalawa	Female	Museum	2007
GWM-M35	Udawalawa	Male	Museum	2007
GWM-M36	Udawalawa	Male	Museum	2016
GWM-M37	Bundala	Male	Museum	2017
VP	Peradeniya	Male	Museum	2012
GW 1	Kotamale	Male	Museum	2013
GW 2	Gampola	Female	Museum	2012
Zoo ST	Kegalle	Female	Preserved	2018
YL 1	Yala	Male	Museum	1993
YL 2	Yala	Female	Museum	1961

**Table 2:** Description of body measurements recorded from study specimens [34].

Parameter	Description
Body weight (BW)	Total body weight
Snout length (SL)	From the tip of the nose to the ocular area of the head
Ear length (anthelix–apex) (EL)	From the bottom of the fold behind the anthelix to the apex of the pinna (left and right) [26]
Head length (HL)	From the upper commissure of the nostrils to the nape [35]
Head body length (physiological) (HBL)	From the tip of the nose to the sacrococcygeal joint, following the animal’s contours with the pangolin in a physiological recumbent position
Tail length (TL) [27]	From the sacrococcygeal joint to the tip of the last caudal vertebral or to the last scale tip of the vertebral scale row (upraising the tail from its base [36])
Total body length (TBL)	From the tip of the nose to the tip of the last caudal vertebral or to the last scale tip of the vertebral scale row
Neck circumference (NC) [37]	Measured not too tightly around the caudal portion of the neck
Shoulder circumference/Axillary girth (SC)	Measured behind and around the shoulders [38]/measured with a tape closely around the chest “immediately behind the forelegs” [39]
Body circumference (BC)	Measured around the middle of the stomach area.
Forelimb length (FLL)	The measurement from humeroulnar joint (elbow joint) to the end of the fore footpad on the longest digit/claw of the longest tow.
Hindlimb length (HLL)	Measured from the tip of the calcaneus to the distal end of the footpad on the longest digit/claw of the longest toe [26].
Hindfoot diameter (HFD)	Measured from the starting point of metatarsals to the distal end of the footpad on the longest digit/claw of the longest toe. (both feet) [26]

Adapted from (Perera et al. [34]). Weight is measured to 0.1 kg, all lengths to 0.1 cm.

**Table 3:** Body measurements of *M. crassicaudata* specimens observed for the study.

Specimen	Sex	BW (kg)	SL (cm)	EL (cm)	HL (cm)	HBL (cm)	TL (cm)	TBL (cm)	NC (cm)	SC (cm)	BC (cm)	FLL (cm)	HLL (cm)	HFD (cm)
**Juvenile**														
DV	Female	4.33	4.40	7.30	9.60	29.20	26.40	55.60	16.40	33.10	36.80	8.60	7.30	3.70
Zoo RS	Female	2.30	3.60	6.20	8.40	21.60	24.70	46.30	5.70	13.70	18.40	7.60	6.20	3.15
NB 1	Male	0.06	2.90	0.80	3.90	10.80	6.50	17.30	3.00	6.40	9.40	4.60	2.90	1.45
NB 2	Male	0.08	3.10	0.70	4.60	11.50	7.60	19.10	4.50	7.30	10.20	5.10	3.40	1.70
**Sub-adult**														
GW 1	Female	-	2.10	1.60	10.50	40.40	30.20	70.60	14.90	30.50	41.60	9.70	5.40	3.30
GW 2	Male	-	1.70	1.30	11.50	54.40	42.40	96.80	16.70	39.90	45.10	13.40	8.60	4.70
M34	Female	-	5.30	2.80	17.00	46.50	48.80	95.30	20.30	42.80	50.70	12.00	9.60	5.70
MP 1	Male	6.15	5.20	15.70	13.20	45.90	44.50	90.30	24.00	40.00	52.70	10.50	8.45	5.90
MP 2	Male	5.70	5.00	13.00	15.00	47.00	42.00	89.00	22.30	38.60	48.60	9.80	8.10	5.75
PGIAR	Male	2.40	4.10	6.60	9.00	18.60	28.60	47.20	14.30	30.00	31.00	7.80	6.60	3.60
RS 1	Female	6.50	6.10	12.10	13.30	46.80	41.30	88.10	21.60	40.30	50.30	16.20	12.10	5.45
RS 2	Female	7.33	6.50	12.70	13.60	45.20	47.50	92.70	18.30	39.20	48.30	17.30	12.70	6.10
VP	Male	-	4.50	2.40	13.40	58.50	42.50	101.00	16.40	35.00	43.80	11.70	6.90	6.90
RG	Male	5.15	6.04	2.85	13.00	56.00	37.5	93.50	17.20	-	-	-	8.70	6.47
**Adult**														
HY	Male	20.40	7.80	8.30	16.30	75.30	61.00	136.30	32.10	61.70	73.40	19.70	8.30	5.95
M35	Male	-	4.80	2.90	11.50	65.60	42.80	108.40	17.80	38.20	46.20	17.50	12.30	5.30
M36	Male	-	5.10	2.20	11.90	61.20	39.60	100.80	39.60	43.20	52.90	18.90	12.60	5.30
M37	Male	-	5.90	3.40	12.40	72.70	55.90	128.60	17.50	56.40	66.30	22.40	14.60	4.80
YL 1	Male	-	5.90	2.70	14.90	73.60	52.50	126.10	21.50	47.80	55.40	18.50	11.90	8.85
YL 2	Female	-	5.80	1.90	13.90	62.90	47.80	110.70	24.20	46.20	50.80	17.10	9.30	5.30
KL 1	Male	48.76	12.40	14.00	26.60	85.35	91.45	176.80	42.00	80.80	103.50	16.50	14.00	8.25
KL 2	Male	34.15	9.60	10.20	20.40	81.92	75.58	157.50	37.20	72.00	90.70	12.70	10.20	6.35

**Table 4:** Summary of morphometric parameters of different age categories.

Parameter	Age Category		
	Juvenile (*n* = 5)	Subadult (*n* = 9)	Adult (*n* = 8)
Total length (cm)	≤56.0	56.1–101.0	˃101.0
Body weight (kg)	≤4.3	4.34–7.3	˃7.33
Shoulder circumference (cm)	≤30.0	30.1–43.0	˃43.0
Body circumference (cm)	≤37.0	37.1–53.0	˃53.0
Neck circumference (cm)	≤16.0	16.1–24.0	˃24.0
Tail length (cm)	≤29	29.1–49.0	˃49.0
Head Length (cm)	≤10.0	10.1–17.0	˃17.0
Snout length (cm)	≤4.0	4.1–7.0	˃7.0
Hind foot diameter (cm)	≤4.0	4.1–˃8.0	˃8.0
Tail-to-body length ratio	0.97	0.89	0.79

**Table 5:** Mean number of scales present on body regions of *M. crassicaudata* specimens.

Body Region	Age Classes			Total Scales (*N* = 25)
	Juvenile (*N* = 4)	Subadult (*N* = 9)	Adult (*N* = 12)	
Trunk	130 ± 2	132 ± 3	133 ± 7	132 ± 5
Head and Neck	87 ± 7	86 ± 8	86 ± 6	86 ± 7
Fore Limbs	88 ± 6	88 ± 10	89 ± 9	88 ± 8
Hind Limbs	72 ± 4	74 ± 9	76 ± 5	74 ± 6
Tail	131 ± 5	131 ± 5	129 ± 10	130 ± 7
Total	507 ± 7	510 ± 20	514 ± 7	511 ± 21

**Table 6:** Mean number of scales present on scale rows in body and tail regions of *M. crassicaudata* specimens.

Body Region	Age Classes			Sex	
	Juvenile (*N* = 4)	Subadult (*N* = 9)	Adult (*N* = 12)	Female (*N* = 7)	Male (*N* = 18)
Trunk					
MD	15	16 ± 1	16±1	15±1	15±1
DL 1	15	15	15±1	15	15±1
DL 2	13 ± 1	14 ± 1	14 ± 1	14	14 ± 1
DL 3	13 ± 1	13 ± 1	13 ± 1	13 ± 1	13 ± 1
DL 4	6	7 ± 1	7 ± 1	7 ± 1	7 ± 1
DL 5	5	5	5.00	5	5
DL 6	4	4	4.00	4	4
Tail					
TMD	15 ± 1	16 ± 1	16 ± 1	15 ± 1	15 ± 1
TDL	15 ± 1	16 ± 1	16 ± 1	15 ± 1	15 ± 1
LT	15 ± 1	15 ± 1	15 ± 1	15 ± 1	15 ± 1

**Table 7:** Summary of scale morph/type in *M. crassicaudata* specimens.

Scale Morph	Description	Figure
Broad rhombic/scapular shaped scales	Larger convex shape outer edge. Present on: dorsolateral region of the trunk and tail. The largest scales on the posterior of the body at the proximal end of the tail are broad rhombic type. Length is less than the width measurement. Width/length ratio ≥ 1. Number of grooves: 24 to 51. The average number of scales on a pangolin: 411 ± 20.	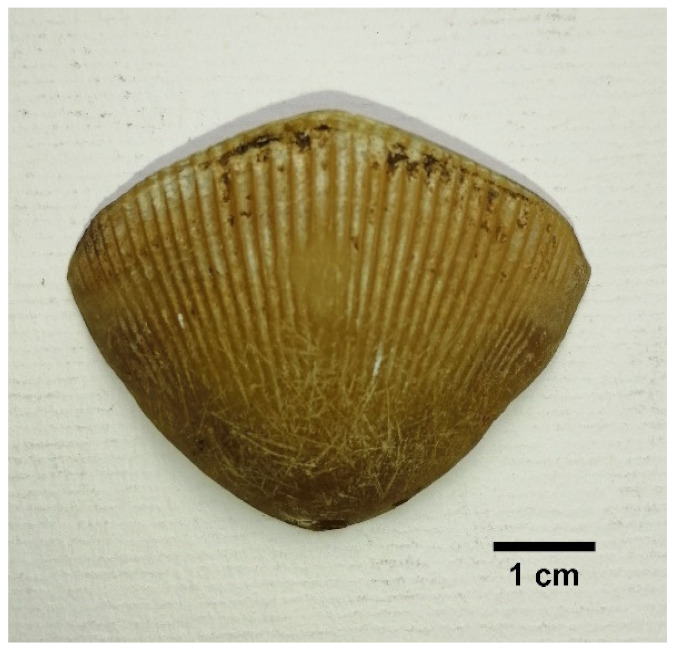
Elongated kite-shaped scale	Horn shaped and flat surface. A derivative of broad rhombic shaped scales. The outer edge has a lower convex/ acute angle. Present on: scapular region, neck region, and forelimbs. Width/length ratio is <1. Number of grooves: 15 to 33. The average number of scales on a pangolin: 69 ± 4.	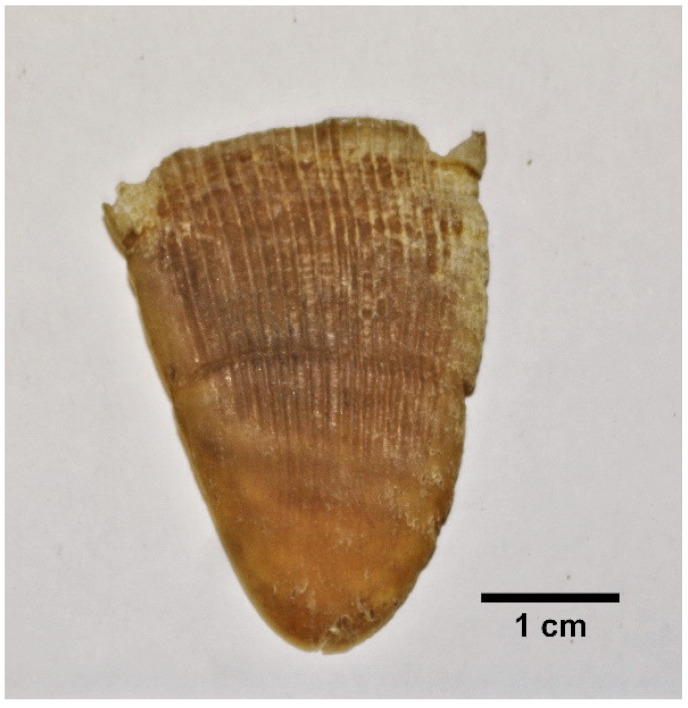
Folded shaped scales	Sharply pointed scales with the tip slightly bent towards left or right, depending on the orientation. Covers both dorsal and ventral part of the tail. The dominant scale on lateral rows (outer edges) of the tail. Dorsal width/ventral width ratio is ≤1. Number of grooves: 20 to 43. The average frequency on a pangolin: 31 ± 3.	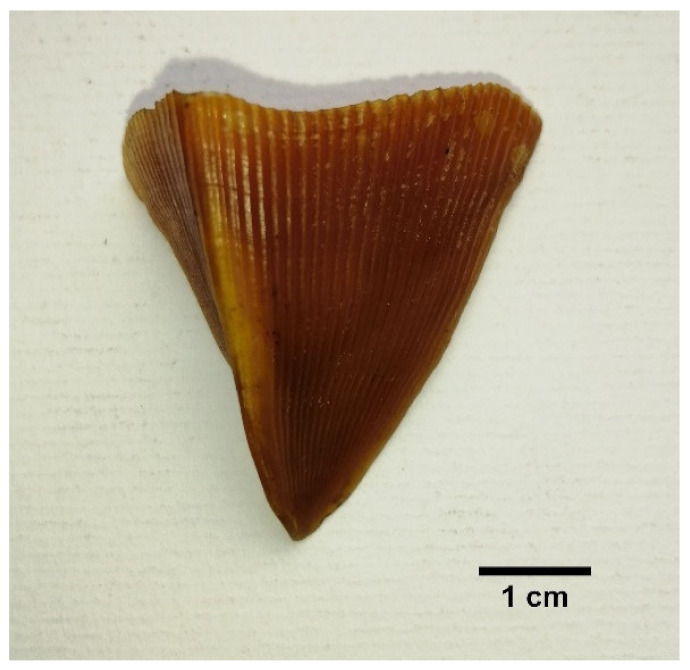

## Data Availability

Data is contained within the article.
